# Editorial: Infection, Inflammation, Cardiovascular Diseases, and Neurodegeneration

**DOI:** 10.3389/fnins.2021.750172

**Published:** 2021-09-09

**Authors:** Deng-Feng Zhang, Xian-Le Bu, Gjumrakch Aliev, Feiqi Zhu

**Affiliations:** ^1^Key Laboratory of Animal Models and Human Disease Mechanisms, Kunming Institute of Zoology, Chinese Academy of Sciences, Kunming, China; ^2^Department of Neurology and Centre for Clinical Neuroscience, Daping Hospital, Third Military Medical University, Chongqing, China; ^3^GALLY International Biomedical Research Institute, San Antonio, TX, United States; ^4^Department of Neurology, The Third Affiliated Hospital, Shenzhen University, Shenzhen, China

**Keywords:** infection, inflammation, neurodegeneration, immune, vasculature

The global burden of neurodegenerative disorders, such as Alzheimer's disease (AD), Parkinson's disease (PD), vascular dementia, and stroke increases rapidly (GBD Neurology Collaborators, 2019). Great efforts are needed to promote the understanding, treatment, and prevention of these diseases. Although remarkable progress have been made in the comprehension of the pathophysiology of neurodegeneration, the etiology is not yet completely understood. Immune system is highly involved in the development of neurodegeneration, since immune genes play a prominent part in genetic risk of typical neurodegenerative diseases (Jonsson et al., 2013; Zhang et al., 2019; Harding et al., 2021). Investigation of immune imbalance, infection and inflammation in neurodegeneration has been a major focus of the field. In this Research Topic, a dozen papers has been compiled, covering multiple aspects regarding inflammation in neurodegeneration.

Microglia activation has been a major process during the development of neurodegeneration. Understanding the activation and regulation of microglia is essential for the management of neuroinflammation. Singh et al. showed that Japanese encephalitis virus (JEV) infection increases chemokines (C-C motif) receptor 2 (CCR2) mediated microglia activation. CCR2 inhibition reduces microglia activation and neurotoxic proinflammatory mediators after JEV infection. Their results provided an important therapeutic target for regulating the microglia activation in pathogenesis of neurodegenerative diseases. In addition to virus infection, bacterial infection such as Chronic Periodontitis (CP) was supposed to be involved in the development of AD (Kamer et al., 2015; Chen et al., 2017). To explore the cross-links between periodontitis and AD, Rong et al. analyzed serum proteins from CP patients and controls using the two-dimensional differential in-gel electrophoresis proteomic approach. They found that 10 serum proteins including cathepsin B (CTSB) were altered in CP patients, and serum concentration of CTSB has a negative correlation with cognition. Importantly, in human neuroblastoma SK-N-SH cells overexpressing wild-type APP695, Porphyromonas gingivalis treatment increases the protein level of CTSB in a TNF-α dependent manner. The increase of CTSB then leads to a prominent increase in Aβ_1−40_ and Aβ_1−42_ in the cell lysates, which could be rescued by CTSB inhibition. Their results provided a TNF-α-CTSB based molecular mechanism underlying the increased risk of AD in CP patients.

Besides pathogens, it has been demonstrated that neurological changes correlated with changes in the gut microbiota. Jeon et al. investigated the changes in the gut microbiota composition and diversity of the middle cerebral artery occlusion ischemic stroke pig model. They found elevated systemic inflammation and reduced microbial diversity at acute stage of stroke. For instance, abundance of the Proteobacteria was significantly increased, while Firmicutes and Lactobacillus decreased poststroke. Interestingly, the microbial pattern of poststroke model returned to prestroke-like status at late stage of stroke, suggesting the plasticity of gut microbiome in the course of stroke. Their findings might benefit the assessment of stroke pathology and the development of therapeutic targets.

Systemic inflammation might be the potential link between infection and neurological alterations, as indicated by Yan et al. and Liu et al.. The long-term administration of levodopa (L-dopa), the gold-standard treatment for PD, may lead to L-dopa-induced dyskinesia (LID). Yan et al. investigated how systemic inflammation exacerbates LID. They constructed a LID rat model under stimulation of lipopolysaccharides (LPS) and long-term treatment of L-dopa. Dyskinesia-related phenotypes, glia activation, and inflammatory responses were then evaluated at several time points. They found that the LPS injection activated the glia and inflammatory response in the striatum of LID rats, and the systemic inflammation exacerbated the intensity of abnormal involuntary movements induced by L-dopa treatment. Inhibition of the inflammatory response improved the behavioral dysfunction. This study suggests that control of systemic inflammation is crucial for management of drug reaction and complications in neurodegenerative diseases. In another study, Liu et al. assessed the effect of LPS-induced systemic inflammation on brain functional connectivity in aged rats. They found that rats with intraperitoneal injected LPS showed higher levels of inflammatory cytokines and astrocyte activation in the hippocampus, which leads to decreased functional connectivity on the right orbital cortex, right olfactory bulb, and left hippocampus, and finally impaired memory performance. Inhibition of the inflammatory activation in the hippocampus improved the memory performance and functional connectivity. These data demonstrated that inhibiting neuroinflammation improves the brain functional connectivity.

Sepsis-associated delirium (SAD) is another good model for investigating the interaction between brain dysfunction and inflammatory responses. Li et al. performed a retrospective study analyzing the association between SAD and lymphocyte counts in the peripheral blood, alongside a prospective trial evaluating the predictive value of lymphocyte for early diagnosis of SAD. In the retrospective analysis, they showed that lymphocyte counts in the SAD group were significantly higher than in non-delirious counterparts. Importantly, they found that an NK cell count cut-off value of 87 cells/ml in septic patients at ICU admission was predictive of delirium with a sensitivity of 80.2% and specificity of 80.8%. These preliminary but promising data showed the value of measuring peripheral lymphocyte for monitoring the pathophysiological process in the central nerve system.

The blood-brain barrier (BBB), which is consisted of vascular, immune, and neural cells, is the key for the interaction between immune system and the central nervous system. BBB dysfunction are frequently observed in the pathology and progression of neurological diseases (Xiao et al., 2020). Since elevated pulse pressure can cause BBB dysfunction that may drive or contribute to adverse neurological changes, Levin et al. reviewed the impact of elevated pulse pressure on microvascular damage and discussed efforts to repurpose blood pressure medications to prevent or treat dementia. They proposed that new drugs or devices should be developed to safely reduce elevated pulse pressure specifically to the brain. As indicated by Levin et al., vascular risk factors such as hypertension and diabetes are associated with cognitive decline and the risk of dementia (Purnell et al., 2009). To investigate whether the aggregation of vascular risk factors modulates the spontaneous brain activity, Zhuang et al. performed a resting-state functional MRI scanning of patients with mild cognitive impairment (MCI). They found that MCI patients with high vascular risk showed decreased amplitude of low-frequency fluctuation in the left hippocampus compared to that of healthy subjects with high vascular risk. This preliminary data highlighted a potential imaging mechanism underlying vascular contribution to brain activity.

As shown above, cytokines such as interleukins and chemokines such as CCR2 play pivotal and comprehensive roles in neurodegenerative diseases, however, the exact activating pathways and cell-type specificities of such inflammatory molecules remained unclear. Chen et al. reviewed the way how microglia, astrocyte, and oligodendrocyte respond to a typical interleukin, the IL-17A, and summarized plant compounds targeting IL-17A. Considering the complicated activation and regulation pattern of cytokines, more focused and fine-grained examination of the exact roles of cytokines in neurodegenerative diseases is needed.

The pathogen hypothesis, which postulates a causal role of infectious agents (e.g., herpes virus) in the development of AD, has been a non-negligible topic, whereas amyloid beta is recognized to be an antimicrobial peptide that protects against fungal, bacterial, and viral infection (Gosztyla et al., 2018). However, it is unclear when the hypothesis will benefit the clinical practice. Nevertheless, it is time to “Connect the Dots,” as proposed by Ethan R. Roy and Wei Cao from Baylor College of Medicine. In their Perspective, they summarized the actions and roles of Aβ in AD neuropathogenesis, especially in the microbial entrapment related processes. They also described the activation of the IFN antiviral pathway and depict a multilayered connection between antiviral immune response and other agents and factors relevant to AD. Regardless of the unclear molecular mechanism underlying the antiviral immune response in both the CNS and peripheral system, taking anti-infection into consideration for the development of AD therapy might be a crucial step.

In summary, the articles and reviews in this Research Topic covered multiple aspects including key molecules, pathways, cell types, and brain regions in typical neurodegeneration regarding viral and bacterial infection, gut microbiota, and systemic inflammation. These scattered but diverse studies broadened the boundary of discipline of neuroinflammation. Nevertheless, there are much broader unknown area regarding inflammation and neurodegeneration. Hopefully, more and more studies and projects are ongoing to uncover the intricate relationship between immunogical and vascular factors and neurodegenerative disorders.

## Author Contributions

All authors listed have made a substantial, direct and intellectual contribution to the work, and approved it for publication.

## Funding

This work was supported by the National Natural Science Foundation of China (82022017 and 31970965), the Youth Innovation Promotion Association of Chinese Academy of Sciences, the Applied Basic Research Foundation of Yunnan Province (2019FA009 and 2019FI015), the Sanming Project of Medicine in Shenzhen (SZSM201801014), and the Key project of Shenzhen Science and Technology Innovation Committee (JCYJ20200109143431341).

## Conflict of Interest

The authors declare that the research was conducted in the absence of any commercial or financial relationships that could be construed as a potential conflict of interest.

## Publisher's Note

All claims expressed in this article are solely those of the authors and do not necessarily represent those of their affiliated organizations, or those of the publisher, the editors and the reviewers. Any product that may be evaluated in this article, or claim that may be made by its manufacturer, is not guaranteed or endorsed by the publisher.
